# Genome-wide analysis identifies a role for common copy number variants in specific language impairment

**DOI:** 10.1038/ejhg.2014.296

**Published:** 2015-01-14

**Authors:** Nuala H Simpson, Fabiola Ceroni, Rose H Reader, Laura E Covill, Julian C Knight, the SLI Consortium, R Nudel, A P Monaco, E Simonoff, P F Bolton, A Pickles, V Slonims, K Dworzynski, A Everitt, A Clark, J Watson, J Seckl, H Cowie, W Cohen, J Nasir, D V M Bishop, Z Simkin, Elizabeth R Hennessy, Patrick F Bolton, Gina Conti-Ramsden, Anne O'Hare, Gillian Baird, Simon E Fisher, Dianne F Newbury

**Affiliations:** 1 Wellcome Trust Centre for Human Genetics, University of Oxford, Oxford, UK; 2 University Child Health and DMDE, University of Aberdeen, Aberdeen, UK; 3 Departments of Child and Adolescent Psychiatry, Social, Genetic and Developmental Psychiatry Centre, Institute of Psychiatry, King's College London, London, UK; 4 School of Psychological Sciences, University of Manchester, Manchester, UK; 5 Department of Reproductive and Developmental Sciences, University of Edinburgh, Edinburgh, UK; 6 Children's Neurosciences Department, Evelina Children's Hospital and King's Health Partners, London, UK; 7 Max Planck Institute for Psycholinguistics, Nijmegen, The Netherlands; 8 Donders Institute for Brain, Cognition and Behaviour, Radboud University, Nijmegen, The Netherlands; 9 St John's College, University of Oxford, Oxford, UK

## Abstract

An exploratory genome-wide copy number variant (CNV) study was performed in 127 independent cases with specific language impairment (SLI), their first-degree relatives (385 individuals) and 269 population controls. Language-impaired cases showed an increased CNV burden in terms of the average number of events (11.28 *vs* 10.01, empirical *P*=0.003), the total length of CNVs (717 *vs* 513 Kb, empirical *P*=0.0001), the average CNV size (63.75 *vs* 51.6 Kb, empirical *P*=0.0005) and the number of genes spanned (14.29 *vs* 10.34, empirical *P*=0.0007) when compared with population controls, suggesting that CNVs may contribute to SLI risk. A similar trend was observed in first-degree relatives regardless of affection status. The increased burden found in our study was not driven by large or *de novo* events, which have been described as causative in other neurodevelopmental disorders. Nevertheless, *de novo* CNVs might be important on a case-by-case basis, as indicated by identification of events affecting relevant genes, such as *ACTR2* and *CSNK1A1*, and small events within known micro-deletion/-duplication syndrome regions, such as chr8p23.1. Pathway analysis of the genes present within the CNVs of the independent cases identified significant overrepresentation of acetylcholine binding, cyclic-nucleotide phosphodiesterase activity and MHC proteins as compared with controls. Taken together, our data suggest that the majority of the risk conferred by CNVs in SLI is via common, inherited events within a ‘common disorder–common variant' model. Therefore the risk conferred by CNVs will depend upon the combination of events inherited (both CNVs and SNPs), the genetic background of the individual and the environmental factors.

## Introduction

Specific language impairment (SLI) is a developmental disorder that, in the absence of neurological deficits, affects an individual's spoken and/or receptive language acquisition. SLI is a common but genetically complex disorder with an estimated prevalence of up to 7%^1^ and shows significant overlap with autism, dyslexia and ADHD, both phenotypically^2^ and genetically.^3, 4^ Like many common disorders, the majority of the genetic risk for SLI is expected to be conferred by combinations of common genetic variants that is, the ‘common disorder–common variant' model.^5^ Nonetheless, a growing body of evidence suggests that single nucleotide variants alone do not explain the heritability of complex traits (the ‘missing heritability') and that the underlying aetiology may include other factors such as copy number variants (CNVs), rare variants and epigenetic modifications.^6^ Studies have found that individuals with autism or ADHD generally have an increased burden of rare CNVs compared with controls^7, 8, 9^ and that the severity of phenotype across neurodevelopmental disorders may be positively correlated with the burden of large CNVs.^10^ The ‘burden' of CNVs can be considered in many ways, for example, the number of CNVs an individual carries, the average size of CNVs, the total size of CNVs across the genome or the number of genes affected by CNV events. Similarly, one can filter the types of CNVs considered, restricting the investigation to rare, *de novo*, exonic or large (usually defined as >1 Mb in the literature) events. Individuals with autism from simplex families (ie, parents and a single affected child) have been reported to carry a higher rate of *de novo* CNVs than those from multiplex families (ie, parents and multiple affected children).^11, 12, 13^ Some CNVs have been associated across disorders; for example, a 600 kb microduplication on 16p11.2 has been associated with childhood apraxia of speech,^14, 15^ autism,^16^ bipolar disorder and schizophrenia,^17^ indicating that the same CNV may give different outcomes. The exact outcome has been proposed to depend on the genetic background of an individual and environmental cues. Other CNVs are not recurrent within a disorder but private to a particular family, presumably contributing to a biological pathway that is shared in other individuals.

We explore the contribution of CNVs to SLI, by studying a set of families collected by the SLI Consortium (SLIC). We compare CNV burden between independent cases and unselected population controls and examine CNV load across the wider SLIC sample set, which includes first-degree relatives of variable affection status.

## Materials and methods

### Inclusion criteria for SLI samples

One-hundred and twenty-seven independent cases with SLI (92 males and 35 females) and 385 available first-degree relatives (parents and siblings (sibs), 192 males and 193 females from 152 families) from the UK-based SLIC were analysed for CNVs. This cohort has previously been described in detail.^18, 19, 20, 21^ The SLIC cohort consists of British nuclear families ascertained to include at least one child affected by SLI, defined as expressive and/or receptive language skills (ELS and RLS, respectively) ≥1.5 SD below the normative mean and nonverbal IQ not >1.5 SD below that expected for their age (77.5). Language skills were measured in all children using the Clinical Evaluation of Language Fundamentals (CELF-R);^22^ a battery of language-based tests that assess a range of traits and thus provide a broad profile of language ability in the child. Nonverbal skills were measured by the WISC Perceptual Organisation Index (a composite score derived from Picture Completion, Picture Arrangement, Block Design and Object Assembly subtests).^23^ In the current study, independent cases were selected to represent one affected individual (as defined above) per family. All available first-degree relatives (parents and siblings) were then used to follow-up findings that were significant in the independent cases. For these follow-up analyses, siblings were classified as affected (as defined above, 37 individuals), unaffected (ELS and RLS above mean, 19 individuals) or undefined language status (if they did not meet the criteria for affected or unaffected or had missing CELF data, 105 individuals). For parents, CELF-R data were not available. However, we were able to classify parental language status using a test of non-word repetition (NWR), which has been proposed as a strong behavioural marker of SLI^24, 25^ and shows high sensitivity and specificity of a positive history of language difficulties in adult subjects.^26^ Thirty-five parents were classified as affected (NWR >1.5 SD below the mean), 27 were unaffected (NWR >mean) and 162 had undefined language status (did not meet the criteria for affected or unaffected, or were missing NWR data). In our child cohort, the NWR measure was observed to have a moderate level of sensitivity (45% of affected children had NWR scores below −1.5 SD) and a high specificity (none of the unaffected sibs had NWR scores below −1.5 SD). Thus, although we expect the NWR measure to classify some parents with a positive history of language impairment as unknown, importantly, it is less likely to classify unaffected parents as affected. Ethical agreement for the SLIC study was given by local ethics committees, and all subjects provided informed consent.

### Control samples

Two-hundred and sixty-nine healthy ‘white-British' adult population controls (115 males and 154 females), unselected in terms of language ability, were obtained from a study of gene expression in primary immune cells.^27^ The study was approved by the Oxfordshire Research Ethics Committee (COREC reference 06/Q1605/55).

### SNP genotyping

DNA was extracted from peripheral blood or buccal smears and all samples were genotyped on the Illumina HumanOmniExpress-12v1 Beadchip (San Diego, CA, USA) that contains ~750 000 SNPs. SNPs were excluded if the gentrain (genotype clustering quality) score was <0.5 or genotyping success rate was <95%. Samples were excluded if they had <95% SNP genotype rate, or heterozygosity rate of ≥±2 SD or fell outside the European cluster in a principal components analysis. Importantly, all samples were genotyped on the same arrays.

### CNV calling

CNVs were identified using PennCNV (16 June 2011 version)^28^ and QuantiSNP (v2.2).^29^ For both algorithms, CNVs were required to have at least three consecutive SNPs and a confidence value (PennCNV) or log Bayes Factor (QuantiSNP) of >10. In PennCNV individuals with an SD for the log R ratio (LRR) >0.35, a B-allele frequency (BAF) drift value >0.002 or a waviness factor >0.04 or <−0.04 were excluded. In QuantiSNP, individuals with an average SD for the LRR >0.3 or an SD for BAF >0.15 were excluded.

If a CNV was predicted by both PennCNV and QuantiSNP, with a minimum intersection of 50% each way, it was considered to be of ‘high confidence' and was carried forward for analyses described below. The innermost boundaries of the two algorithm calls were used. CNVs were excluded if they spanned the centromere or telomeres.

### Rare, novel and *de novo* CNV identification and validation

All ‘high-confidence' CNVs were compared against the Database for Genomic Variants (DGV; downloaded from UCSC genome browser hg19, January 2012) to identify ‘rare and novel' CNVs. Those that intersected <50% with five or less CNVs in the DGV were considered rare. Those that did not overlap with any CNV in the DGV were classed as novel.

To detect *de novo* CNVs, 161 individuals (67 probands, 18 affected siblings, 27 unaffected siblings and 49 siblings of undefined affection status) who had genotype data available for both parents were analysed using trio and quartet algorithms in PennCNV.

All rare events >100 kbp, all novel exonic events >100 kbp and all *de novo* exonic events were subsequently validated by quantitative PCR using four PCR primer pairs, two outside the CNV and two within it. PCRs were performed in triplicate using iQ SYBR Green Supermix (Bio-Rad, Hercules, CA, USA) and calibrated against a control DNA that did not contain the identified CNVs and a control gene (*ZNF423*) that did not contain any CNVs within our sample set. The parents of individuals with *de novo* exonic CNVs were also examined. Copy numbers in each individual were calculated using the 2^−ΔΔCt^ method.^30^

### Statistical analysis of CNV burden

‘High-confidence' CNVs, as defined above, were analysed using PLINK v1.07^31^ to identify burden differences between independent cases and population controls. Metrics that differed significantly (empirical *P*<0.05) were then also examined in the first-degree relatives. Burden analyses were also performed for ‘rare and novel' and the *de novo* CNVs. Empirical *P*-values were calculated using 10 000 permutations within PLINK.

PLINK was also employed to determine whether pre-defined gene sets showed enrichment for CNVs in independent cases compared with population controls. Given the phenotypic and genetic links reported between autism and SLI, we specifically interrogated 531 autism-candidate genes (compiled from Xu *et al.*^32^and Betancur *et al.*^33^ and the SFARI database (October 2012)). In addition, we investigated 1315 putative targets of the Foxp2 protein (as reported in Vernes *et al.*^34^). Mutations of *FOXP2* cause developmental language disorder, and targets of this transcription factor have been implicated in language and developmental disorders.^15, 35, 36^

Five candidate regions that have consistently been associated with neurodevelopmental disorders were also interrogated for CNV events and compared between independent cases (127 individuals) and population controls (269 individuals). These consisted of chromosomes 7q11.23,^37^ 15q11-13,^15, 16, 17, 38^ 16p11.2,^38, 39^ 16p13.1^38^ and 22q11.2.^38, 39^

### Pathway analysis

WebGestalt^40^ was used to identify gene ontology (GO) terms (Gene Ontology, version 1.2, 11 November 2012) that were enriched for genes present within ‘high-confidence' CNVs and the ‘rare and novel' CNVs between independent cases and population controls. GO categories that were enriched in the independent cases, but not the population controls, are reported. *P-*values were adjusted for multiple testing using the false discovery rate.

## Results

### Burden analysis

1432 ‘high-confidence' CNVs were identified in 127 independent cases (11.3 per individual), compared with 4081 in 385 SLIC first-degree relatives (10.6 per individual) and 2693 in 269 population control samples (10.01 per individual). A full list of all ‘high-confidence' CNVs identified in SLI cases and their first-degree relatives has been submitted to DGVa (accession estd218).

Four burden metrics (average number of CNVs, average total length of CNVs, average size of CNVs and average number of genes spanned) differed significantly between independent cases and population controls (Table 1). The average number and average total length of CNVs were driven by deletion events (Table 1) while the other two categories were significant for both deletions and duplications (Table 1). SLIC first-degree relatives (who included affected, unaffected and undefined parents and siblings) also had significantly more CNVs that were, on average, longer and covered more genes, than those observed in population controls (Table 1). The same patterns were seen when the first-degree relative sample set was restricted to include only affected, or only unaffected relatives, although the trends did not always reach significance in these smaller sample sets (Table 1). In order to explore the effect of case ascertainment method (currently based upon expressive and receptive language skills (ELS and RLS, respectively) and nonverbal IQ – see Materials and methods) upon the observed trends, we applied an alternative definition of SLI affection within our case cohort. When independent cases were alternatively selected to have NWR scores <1.5 SD below that expected for their age (59 individuals, 46% of cases), the same four burden metrics (average number of CNVs, average total length of CNVs, average size of CNVs and average number of genes spanned) again differed significantly between independent cases and population controls (Table 1).

### Rare and novel CNVs

Approximately 10% of the ‘high-confidence' CNVs were ‘rare and novel'. A total of 131 ‘rare and novel CNVs' were identified in independent cases (1.03 per individual), 319 in SLIC first-degree relatives (0.83 per individual) and 275 in population controls (1.02 per individual; Table 2). The burden of ‘rare and novel' CNVs, for the main part, did not differ significantly between independent cases and population controls (Table 2). Although independent cases had an increased length of duplications than population controls (Table 2), these differences were less significant than those found for all ‘high-confidence' events.

Twenty ‘rare' or ‘novel' CNVs that were larger than 100 kbp were identified in the independent cases, 14 (70%) of which were exonic (Table 3), while 36 were identified in the population controls, of which 23 (64%) were exonic. The rarity of these events precludes a statistical evaluation. However, as a note of interest, these CNVs included the *NDUFB3, NIF3L1, PPEF2, CACNA2D1* and *GPC5* genes, which are expressed in the brain and/or have been implicated in neurological disorder.

### Gene enrichment analysis

No enrichment for autism-candidate genes or Foxp2 targets was observed for the ‘high-confidence', ‘rare and novel' or *de novo* CNVs in independent cases.

### Pathway analysis

There were 719 genes that had GO categories defined within the ‘high-confidence' events in independent cases and 757 within population controls. For the ‘rare and novel' CNVs, 179 genes had GO categories defined within the independent cases and 176 in population controls. Pathway analyses indicated that six GO categories were significantly and specifically enriched in independent cases but not in population controls after correcting for multiple testing. ‘Acetylcholine binding' (GO:0042166, *CHRNA7, CHRNA3, ACHE* and *CHRNB4*), ‘cyclic-nucleotide phosphodiesterase activity' (GO:0004112 and GO:0004114, *PDE8A, PDE1A, PDE4D, PDE6H* and *PDE1C*) and ‘MHC protein complex' (GO:0042611, *HLA-DMA, HLA-C, HLA-H, HLA-DQA1, MICA* and *HLA-DMB*) were enriched when considering all CNV events. While the cellular components ‘proteasome activator complex' (GO:0008537, *PSME1* and *PSME2*) and ‘nuclear inclusion body' (GO:0042405, *NXF1* and *ATXN1*) were enriched in the ‘rare and novel' CNV set (Table 4).

### *De novo* CNVs

Genotype data were available for both parents for 161 children (including 85 affected individuals (independent cases or affected siblings), 27 unaffected siblings and 49 individuals of undefined affection status). Analyses of these trios/quartets identified 77 putative *de novo* CNVs in 56 individuals, of whom 24 were affected (17 independent cases), 12 were unaffected and 20 had undefined affection status. Although the sample size is small, burden analysis comparisons did not find differences in the rate or size of *de novo* CNVs between the affected and unaffected individuals.

Genic *de novo* CNVs in independent cases (5 events in 4 individuals; Table 3) were all confirmed to be absent in the parents by qPCR. Four of the five events were not observed in any other individuals in this dataset. Three of these include genes of potential interest for SLI (see Discussion). Two of the *de novo* CNVs fell within regions of known structural variation in neurodevelopment; 8p23.1^41^ and 22q11.2,^38, 39^ although they were smaller than the typical micro-deletion/-duplication events typically reported.

### Specific candidate regions

Five CNV candidate regions in neurodevelopmental disorders were investigated: 7q11.23, 15q11-13, 16p11.2, 16p13.1 and 22q11.2. No CNVs were found in 7q11.23, 16p11.2 or 16p13.1 in independent cases. CNVs on 15q11-13 and 22q11.2 were found in both independent cases and population controls (Supplementary Table). The frequency of these events was similar between independent cases and population controls (Supplementary Table). All the events identified consisted of small CNVs within these sites rather than the classical large events typically associated with neurodevelopmental disorder.

## Discussion

An exploratory study of CNVs in individuals with SLI and their first-degree relatives was performed. Consistent, statistically significant increases in burden were found for individuals with SLI suggesting that copy number does have a role in this disorder. More specifically, our cases showed a significantly higher number of deletions, with larger CNVs and deletions that covered more genes than controls. The differences in burden appear to be primarily driven by the size of events. Our cases, on average only carried one more CNV than population controls, but each event was, on average, 12 kb longer and the total CNV length across the genome therefore totalled 200 kb more in cases than population controls. Furthermore, these events on an average, hit four more genes in the cases than population controls.

In contrast to that reported for autism and ADHD^7, 8, 9^ we found only an increase in the average total length of ‘rare and novel' duplications and the average ‘rare and novel' duplication size in independent cases compared with population controls (Table 2). Furthermore, we note that the majority of CNVs observed in independent cases were <100 kb. Sizeable events are reported to be of importance in intellectual disability^10^ but, interestingly, not in developmental dyslexia.^10^ Note, however that the contribution of smaller, common CNVs to dyslexia has yet to be evaluated and that the number of these events in our cohort was small.

We extended our investigations to consider CNV burden across the first-degree relatives of our independent SLI cases. We studied all available parents and siblings (regardless of their language status) as well as subsets of only affected or only unaffected relatives. We again observed a significantly increased burden of larger, genic CNVs compared with population controls (Tables 1 and 2). Furthermore we found that these trends were consistent across the first-degree relatives, regardless of affection status (Tables 1 and 2).

*De novo* CNVs have been reported to be of particular importance in neurodevelopmental disorders, especially when fecundity is reduced. Although our sample set was small, we observed a similar level of *de novo* CNVs across 85 SLI cases and 27 unaffected siblings. Thus, unlike that reported for autism and schizophrenia,^11, 37^ we propose that *de novo* CNVs do not represent a major risk factor for SLI.

Given the data generated from this study, we hypothesise that the increased copy number burden observed in SLI occurs via a ‘common disease–common variant' model in which certain combinations of common CNV events confer the majority of CNV-based risk. In this small sample set, we find evidence that children affected by SLI carry a higher burden of common CNVs of moderate size that hit genes more often than that observed in population controls. This finding extends to the first-degree relatives of children affected by SLI, indicating that the major driving force is likely to be inherited rather than *de novo*. We did not observe a significant correlation between CNV burden and language-related phenotypic scores (Supplementary Figure 1) amongst cases and their first-degree relatives (Supplementary Figure 1), indicating that the correlation between CNV burden and absolute risk is not straightforward. Together, these data suggest that the absolute risk conferred by CNVs depends upon the position and combination of events inherited, and the genetic background of the individual, which may also include sequence variants of effect and environmental factors.

Although we did not observe an increased rate of *de novo* CNVs in cases, we do not preclude the possibility that these events are important on a case-by-case basis. A number of genes within *de novo* CNVs represent interesting candidates (Table 3). A deletion in the *ACTR2* gene was found in an independent case (SLI-45_2, Table 3) and his monozygotic twin, who was also affected, indicating that this event occurred prior to the division of the blastocyst. *ACTR2* encodes a component of the ARP2/3 complex, a reduction of which may cause abnormal neuronal and glial migration and impaired neurite extension.^42^ One independent case (SLI-146_3, Table 3) was found to have two *de novo* events; a deletion in *CSNK1A1*, which has been related to dopamine signalling and ADHD^43^ and a duplication within the region typically duplicated in 22q11.2 microduplication syndrome. A further case (SLI-59_3, Table 3) had a duplication that fell within the 8p23.1 duplication syndrome region, which can include language delay.^41^ Interestingly, each of the independent cases carrying *de novo* CNVs were from simplex families, apart from the monozygotic twin described above, perhaps indicating a different mechanism of risk within isolated cases of language impairment and suggesting that clinical screening of such cases may prove fruitful.

Pathway analyses identified several GO categories of functional interest, six of which survived multiple testing (GO:0004112, GO:0004114, GO:0042166, GO:0042611, GO:0008537 and GO:0042405; Table 4). Acetylcholines (GO:0042166) act as neurotransmitters and cyclic-nucleotide phosphodiesterase enzymes (GO:0004112 and GO:0004114) are widely expressed in brain tissue.^44^ The MHC loci (GO:0042611), *HLA-C* and *HLA-DQA1,* have been recently associated with SLI.^45^ Proteasome activator complexes (GO:0008537) have been associated with neurodegenerative and autoimmune diseases^46^ as have genes in the ‘nuclear inclusion body' GO category (GO:0042405; *NXF1* and *ATXN1)*.

In summary, our exploratory study found that children with SLI and their first-degree relatives have an increased burden of moderate-size CNVs (both deletions and duplications) than population controls. However, in contrast to that reported for other neurodevelopmental disorders, we propose that the majority of copy number effects in SLI are conferred by common inherited events. It has previously been proposed that the burden and size of CNVs correlates with the severity of disorder^10^ and our results fit this model. The increased burden observed for our cases is not as extreme as that described for autism and intellectual disability but contrasts with studies of developmental dyslexia, where no increased burden was found. Furthermore, our findings correspond with the prototypical complex disorder model in which multiple events contribute only a small effect upon the overall phenotype. In SLI, unlike autism, it is unusual to observe isolated cases within families and family members often present with other language and/or reading difficulties. Our model therefore suggests that common inherited events that contribute to SLI may be relevant to other language-related disorders such as dyslexia. The risk of an individual is determined by the specific combination of events that hit contributory loci, in combination with other genetic and environmental risk factors. It should be noted that this exploratory study used a relatively small, but well characterised, cohort. Larger sample sizes will be required to confirm the trends observed here. New technologies such as next generation paired-end sequencing will be able to detect CNVs at a higher resolution than is currently possible with SNP genotyping arrays allowing a more detailed picture of the CNV burden in larger sample sets.

## Figures and Tables

**Table 1:** Burden analysis for (a) all CNVs; (b) deletions; (c) duplications in independent cases compared with population controls

	*No. of CNVs*	*Average no. of CNVs per individual*	*Proportion of sample with one or more CNV*	*Average total length of CNVs spanned per individual (kb)*	*Average CNV size (kb)*	*Average no. of genes spanned by CNVs per individual*	*Proportion of CNVs containing at least one gene*	*Average no. of genes per total CNV (kb)*
*Total Burden*								
Independent cases and population controls								
Cases	1432	**11.28**	1	**717.4**	**63.75**	**14.29**	0.95	0.02
Controls	2693	**10.01**	1	**513.9**	**51.55**	**10.34**	0.99	0.02
Empirical *P*-value		**0.003**	1	**0.0001**	**0.0005**	**0.0007**	1	0.95
All SLIC family members and population controls								
Family members	4081	**10.6**		**720.3**	**70.09**	**12.84**		
Controls	2693	**10.01**		**513.9**	**51.55**	**10.34**		
Empirical *P*-value		**0.03**		**0.0001**	**0.0001**	**0.0005**		
Affected SLIC family members and population controls								
Family members	770	10.69		**773.1**	**77.26**	**12.46**		
Controls	2693	10.01		**513.9**	**51.55**	**10.34**		
Empirical *P*-value		0.08		**0.0001**	**0.0001**	**0.02**		
Unaffected SLIC family members and population controls								
Family members	501	10.89		**792.2**	**71.24**	**13.85**		
Controls	2693	10.01		**513.9**	**51.55**	**10.34**		
Empirical *P*-value		0.07		**0.0002**	**0.0001**	**0.005**		
Independent cases selected on the basis of low NWR and population controls								
Cases	674	**11.42**	1	**704**	**60.46**	**12.51**	0.95	0.02
Controls	2693	**10.01**	1	**513.9**	**51.55**	**10.34**	0.99	0.02
Empirical *P*-value		**0.004**	1	**0.0004**	**0.03**	**0.03**	1	0.92
								
*Deletions*								
Independent cases *vs* controls								
Cases	1027	**8.09**	1	**356**	**45.19**	**7.8**	0.92	0.03
Controls	1878	**6.98**	1	**236.4**	**34.77**	**5.6**	0.94	0.03
Empirical *P*-value	—	**0.001**	1	**0.0001**	**0.0003**	**0.0007**	0.86	0.64
All SLIC family members and population controls								
Family members	2995	**7.78**		**344.8**	**45.51**	**7.44**		
Controls	1878	**6.98**		**236.4**	**34.77**	**5.6**		
Empirical *P*-value	—	**0.002**		**0.0001**	**0.0001**	**0.0005**		
Affected SLIC family members and population controls								
Family members	546	7.58		**352.9**	**49.3**	**6.96**		
Controls	1878	6.98		**236.4**	**34.77**	**5.6**		
Empirical *P*-value		0.07		**0.0001**	**0.0002**	**0.04**		
Unaffected SLIC family members and population controls								
Family members	364	**7.91**		**376.2**	**46.81**	**8.59**		
Controls	1878	**6.98**		**236.4**	**34.77**	**5.6**		
Empirical *P*-value		**0.03**		**0.0001**	**0.002**	**0.002**		
								
*Duplications*								
Independent cases *vs* controls								
Cases	401	3.16	0.91	**392.5**	**121.7**	6.44	0.76	0.02
Controls	813	3.02	0.96	**286.4**	**89.41**	4.72	0.86	0.03
Empirical *P*-value	—	0.31	0.99	**0.003**	**0.005**	0.07	1	0.97
All SLIC family members and population controls								
Family members	1072			**393.3**	**129**			
Controls	813			**286.4**	**89.41**			
Empirical *P*-value	—			**0.0004**	**0.0001**			
Affected SLIC family members and population controls								
Family members	223			**442.7**	**124.5**			
Controls	813			**286.4**	**89.41**			
Empirical *P*-value				**0.0009**	**0.004**			
Unaffected SLIC family members and population controls								
Family members	132			**442.6**	**119.6**			
Controls	813			**286.4**	**89.4**			
Empirical *P*-value				**0.01**	**0.03**			

Abbreviations: CNV, copy number variant; NWR, non-word repetition; SLIC, specific language impairment Consortium.

Those metrics that differed significantly between independent cases and population controls were then examined further in affected first-degree relatives and all first-degree relatives compared with population controls. In Table 1, an alternative definition of affection was also explored; independent cases were selected on the basis of NWR >1.5 SD below that expected for their age. Categories in bold had a *P-*value <0.05.

**Table 2:** Burden analysis for (a) ‘rare and novel' CNVs and deletions; (b) duplications in independent cases compared with population controls

	*No. of CNVs*	*Average no. of CNVs per individual*	*Proportion of sample with one or more CNV*	*Average total length of CNVs spanned per individual (kb)*	*Average CNV size (kb)*	*Average no. of genes spanned by CNVs per individual*	*Proportion of CNVs containing at least one gene*	*Average no. of genes per total CNV (kb)*
*Total burden*								
All CNVs in independent cases *vs* controls								
Cases	131	1.03	0.58	102.4	55.42	2	0.41	0.05
Controls	275	1.02	0.63	77.56	47.4	0.99	0.41	0.07
Empirical *P*-value	—	0.47	0.85	0.08	0.13	0.08	0.54	0.59
								
*Deletions*								
Deletions in independent cases *vs* controls								
Cases	61	0.48	0.38	42.47	33.31	0.46	0.24	0.07
Controls	177	0.66	0.46	62.6	46.36	0.52	0.26	0.05
Empirical *P*-value	—	0.98	0.96	0.98	0.95	0.7	0.74	0.21
								
*Duplications*								
Independent cases *vs* controls								
Cases	67	0.53	0.29	**142**	**88.12**	1.5	0.22	0.03
Controls	97	0.36	0.3	**65.95**	**53.72**	0.45	0.18	0.11
Empirical *P*-value	—	0.14	0.59	**0.004**	**0.006**	0.06	0.2	0.86
All SLIC family members and population controls								
Family members	98			**92.23**	**76.44**			
Controls	97			**65.95**	**53.72**			
Empirical *P*-value				**0.03**	**0.02**			
Affected SLIC family members and population controls								
Family members	22			95.71	89.66			
Controls	97			65.95	53.72			
Empirical *P*-value				0.12	0.08			
Unaffected SLIC family members and population controls								
Family members	18			92.32	67.32			
Controls	97			65.95	53.72			
Empirical *P*-value				0.15	0.22			

Abbreviations: CNV, copy number variant; SLIC, specific language impairment Consortium.

As no significant differences were found for the total burden and deletion burden of ‘rare and novel' CNVs, only independent cases *vs* controls are shown in this table.

Those metrics which differed significantly between independent cases and population controls were then examined further in affected first-degree relatives, unaffected first-degree relatives and all first-degree relatives compared with population controls. Categories in bold had a *P-*value <0.05. Although the affected- and unaffected-only family members did not reach significance, similar trends were seen within these smaller groups.

**Table 3:** Rare and novel events >100 kbp and all *de novo* CNVs in independent cases

*Category of CNV*	*Individual*	*Position (hg19)*	*No. of SNPs*	*Confidence score*	*Genes*	*Intron/exon*	*No. of cases with overlap CNVs (%)*	*No. of overlap CNVs in SLIC first-degree relatives (%)*	*No. of overlap CNVs in population controls (%)*	*Overlap CNVs in DGV?*
Rare	SLI-42_2	chr11:g.122455520_122675454*dup*	105	27	*UBASH3B*	Exonic	2 (1.6)	0	0	
*De novo*	SLI-45_2	chr2:g.65486928_66364645*del*	282	691	*ACTR2,SPRED2*	Exonic	1 (0.8)	0	0	Yes
*De novo*	SLI-59_3	chr8:g.9637318_10340111*dup*	298	19	*LOC157627,MIR124-1,MSRA,TNKS*	Exonic	1 (0.8)	0	0	Yes
*De novo*	SLI-63_3	chr4:g.120289042_120381341*del*	6	17	*FLJ14186,LOC645513*	Exonic	1 (0.8)	0	0	Yes
Rare	SLI-71_2	chr7:g.81788703_81926808*del*	53	183	*CACNA2D1*	Exonic	1 (0.8)	1 (0.3)	0	
Rare	SLI-72_2	chr13:g.92408505_92524032*del*	22	70	*GPC5*	Exonic	1 (0.8)	6 (1.6)	0	
Rare	SLI-77_4	chr5:g.123502228_123623533*del*	34	78	*—*	*—*	1 (0.8)	2 (0.5)	0	
Rare	SLI-77_4	chrX:g.64346959_64764336*dup*	16	22	*LAS1L,ZC3H12B*	Exonic	1 (0.8)	0	0	
Rare	SLI-88_3	chr9:g.13774329_13919229*del*	73	257	*—*	*—*	1 (0.8)	2 (0.5)	2 (0.7)	
Novel	SLI-89_2	chr2:g.201766236_201943431*dup*	14	25	*FAM126B,NDUFB3,NIF3L1,ORC2*	Exonic	1 (0.8)	1 (0.3)	0	
Novel	SLI-90_2	chr2:g.201823460_201943431*dup*	10	13	*FAM126B,NDUFB3,ORC2*	Exonic	1 (0.8)	1 (0.3)	0	
Novel	SLI-90_2	chr11:g.91486518_91668678*del*	57	138	*—*	*—*	1 (0.8)	0	0	
Rare	SLI-93_3	chr10:g.17080633_17211383*dup*	61	135	*CUBN,TRDMT1*	Exonic	1 (0.8)	2 (0.5)	0	
Rare	SLI-95_2	chr17:g.19998377_20103560*del*	13	14	*SPECC1*	Exonic	1 (0.8)	1 (0.3)	1 (0.4)	
Novel	SLI-95_2	chrX:g.91230696_91335411*dup*	5	11	*PCDH11X*	Intronic	3 (2.4)	1 (0.3)	1 (0.4)	
Novel	SLI-110_2	chr2:g.98620765_98814054*dup*	47	95	*VWA3B*	Exonic	1 (0.8)	0	0	
Rare	SLI-111_3	chr13:g.46168409_46274638*dup*	23	43	*FAM194B*	Exonic	1 (0.8)	1 (0.3)	0	
Rare	SLI-112_3	chr5:g.15770553_15921693*dup*	28	55	*FBXL7*	Intronic	1 (0.8)	1 (0.3)	0	
Rare	SLI-121_2	chr11:g.122430752_122684597*dup*	120	33	*UBASH3B*	Exonic	2 (1.6)	0	0	
Rare	SLI-141_1	chrX:g.73422412_73564051*dup*	12	16	*FTX,MIR374A,MIR374B,MIR374C,MIR421,MIR545,ZCCHC13*	Exonic	1 (0.8)	0	0	
Rare	SLI-144_3	chrX:g.35827927_36025401*dup*	30	53	*CXorf22*	Exonic	1 (0.8)	1 (0.3)	0	
*De novo*	SLI-146_3	chr5:g.148883634_148903068*del*	8	13	*CSNK1A1*	Exonic	1 (0.8)	0	0	Yes
*De novo*	SLI-146_3	chr22:g.21105255_21463730*dup*	119	185	*AIFM3,BCRP2,CRKL,LOC400891,LZTR1,TUBA3FP,P2RX6,P2RX6P,PI4KA,SERPIND1,SLC7A4,SNAP29,THAP7,THAP7-AS1*	Exonic	1 (0.8)	0	0	Yes
Rare	SLI-148_2	chr15:g.61751304_61870836*dup*	56	116	*—*	*—*	1 (0.8)	0	0	
Rare	SLI-156_3	chr4:g.76712173_76824078*dup*	28	57	*PPEF2,USO1*	Exonic	1 (0.8)	2 (0.5)	0	

Abbreviations: CNV, copy number variant; SLIC, specific language impairment Consortium; SNP, single-nucleotide polymorphism.

Numbers in brackets are frequencies (%).

**Table 4:** Pathway analysis output of GO terms for genes present in all CNVs and the rare and novel CNVs of independent cases

	*GO category*	*No. of reference genes in the category*	*No. of genes in the gene set and also in the category*	*Expected no. in the category*	*Ratio of enrichment*	*Raw* P*-value*	P*-value adjusted for multiple testing*
All CNVs	Molecular function – cyclic-nucleotide phosphodiesterase activity – GO:0004112	25	5	0.7	7.11	0.0006	0.04
All CNVs	Molecular function – acetylcholine binding – GO:0042166	13	4	0.37	10.94	0.0004	0.04
All CNVs	Molecular function – 3′,5′-cyclic-nucleotide phosphodiesterase activity – GO:0004114	24	5	0.68	7.41	0.0005	0.04
All CNVs	Cellular component – MHC protein complex – GO:0042611	38	6	1.09	5.53	0.0007	0.048
Rare and novel CNVs	Cellular component – proteasome activator complex – GO:0008537	3	2	0.02	84.21	0.0002	0.034
Rare and novel CNVs	Cellular component – nuclear inclusion body – GO:0042405	4	2	0.03	63.16	0.0004	0.034

Abbreviations: CNV, copy number variant; GO, gene ontology.

GO categories are listed that did not occur in population controls and survived multiple testing.
