# Supplementary material for: Genome-wide analysis identifies a role for common copy number variants in specific language impairment
Source: Eur J Hum Genet. 2015 Jan 14;23(10):1370–7. doi: 10.1038/ejhg.2014.296 (PMC4592089; doi:10.1038/ejhg.2014.296)
Supplement: Supplementary Figure 1 [file ejhg2014296x1.pdf]

Supplementary Figure S1 - Correlation between phenotypic traits and CNV measures in family cohort (513 individuals).

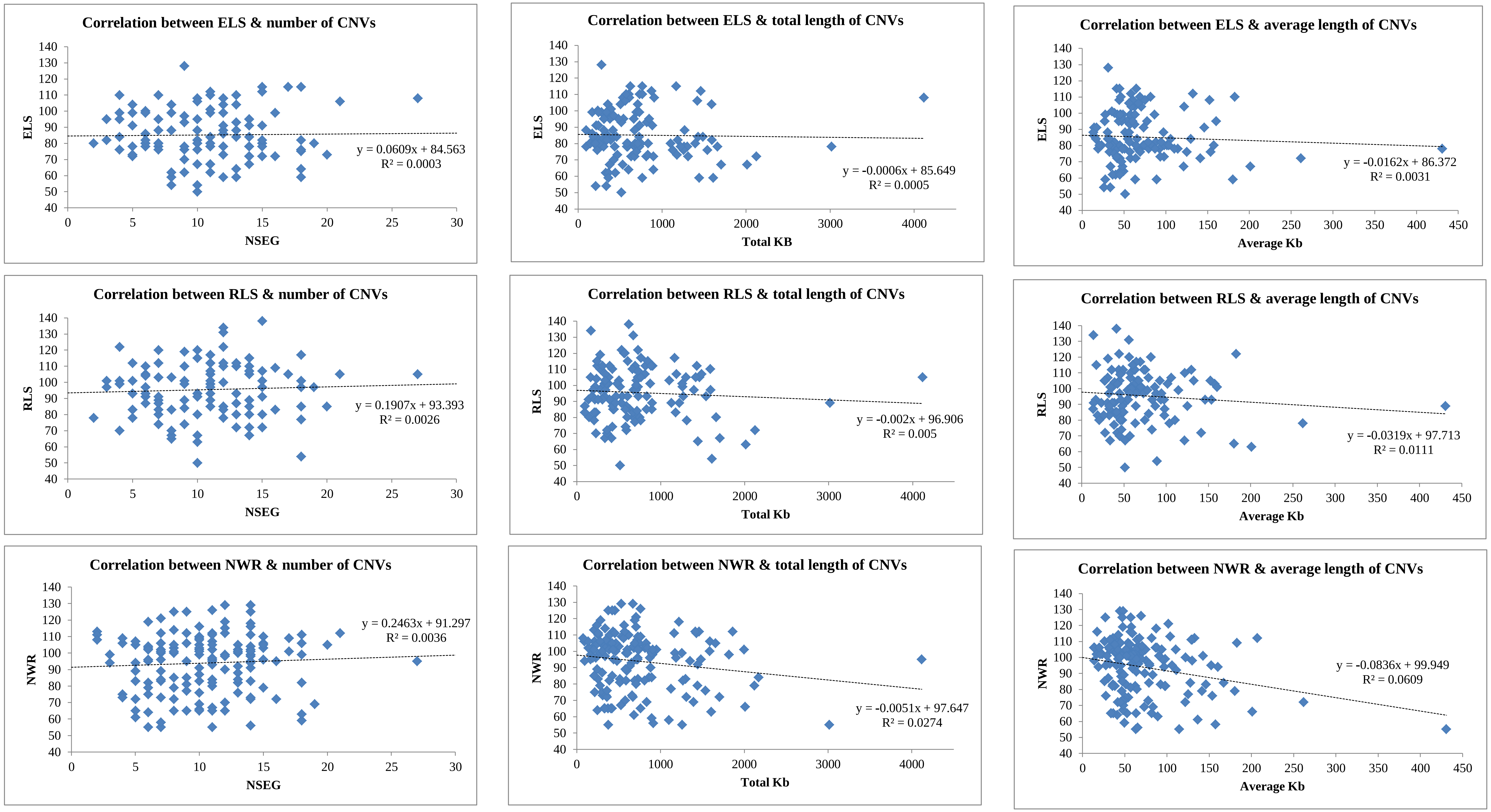

ELS - CELF Expressive Language Score
RLS - CELF Receptive Language Score
NWR - Nonword repetition
