# Supplementary material for: Genome-wide analysis identifies a role for common copy number variants in specific language impairment
Source: Eur J Hum Genet. 2015 Jan 14;23(10):1370–7. doi: 10.1038/ejhg.2014.296 (PMC4592089; doi:10.1038/ejhg.2014.296)
Supplement: Supplementary Table [file ejhg2014296x2.pdf]

Supplementary Table. Copy number changes in regions reported to be of importance in neurodevelopmental disorders and their frequencies in independent cases and population controls.

		No. CNVs in independent SLI cases			No. CNVs in controls				
	Genomic coordinates (hg19)	Total	Deletions	Duplications	Total	Deletions	Duplications	Freq independent cases with CNVs %	Freq controls with CNVs %
7q11.23	chr7:72,023,729 -75,368,283	0	0	0	0	0	0	0	0
chr15q11-13	chr15:19,000,001-33,600,000	20	11	9	37	23	14	15.7%	13.8%
chr15q11.2 (BP1-BP2)	chr15:22.758,000-23,229,000	2	1	1	6	2	4	1.6%	2.2%
16p13.1	chr16:14,752,499-18,792,499	0	0	0	0	0	0	0	0
16p11.2	chr16:28,832,499-30,192,499	0	0	0	0	0	0	0	0
22q11.2	chr22:18,640,000-25,080,000	4	1	3	9	1	8	3.1%	3.3%
